# Plant extracts to enhancing intestinal barrier function in fattening lambs: review

**DOI:** 10.5713/ab.25.0496

**Published:** 2025-10-22

**Authors:** Xiaoyu Niu, Jing Yang, Yuanyuan Xing, Dabiao Li

**Affiliations:** 1 Inner Mongolia Key Laboratory of Animal Nutrition and Feed Science, College of Animal Science, Inner Mongolia Agricultural University, Hohhot, China

**Keywords:** High-concentrate, Intestinal Barrier Function, Lambs, Plant Extracts

## Abstract

The intestine is not only the primary site for nutrient digestion and absorption but also a key defensive organ in animals. An intact intestinal barrier prevents the translocation of antigens, toxins, and pathogens, ensuring animal health. High-concentrate fattening is a common strategy in lamb farming to achieve maximum production performance and economic benefits. However, consuming high-concentrate diets for an extended period of time can damage the intestines of lambs. Plant extracts, characterized by their immunoregulatory, antibacterial, and antioxidant properties, have emerged as potential agents for improving intestinal health. This review comprehensively analyzes the impacts of high-concentrate diets on lamb gut health and systematically summarizes the roles of plant extracts in enhancing intestinal barrier function, and briefly discusses their possible mechanisms of action, aiming to offer scientific support for the rational utilization of plant extracts in the lambs industry.

## INTRODUCTION

In recent decades, the lambs farming industry has undergone remarkable growth. Nevertheless, the scarcity of high-quality roughage resources has emerged as a significant bottleneck, impeding its further expansion. To optimize production performance and maximize economic returns, producers commonly implement high-concentrate feeding strategies for rapid fattening of lambs. However, prolonged consumption of such diets allows excessive bypass starch to enter the small and large intestines, subsequently reducing intestinal pH and elevating lipopolysaccharide (LPS) levels [1–3]. This cascade of events damages intestinal epithelial cell structures [4], disrupts the integrity of intercellular tight junctions [5], and facilitates the translocation of free LPS into the circulatory system [6], as a result, systemic oxidative stress and immune activation occur, causing severe intestinal oxidative damage that compromises animal growth [7,8]. Against the backdrop of stringent “antibiotic restriction” in livestock breeding and “antibiotic prohibition” in feed manufacturing, plant extracts including polyphenols, polysaccharides, flavonoids, and plant essential oil have garnered increasing attention [9,10]. A growing body of evidence, such as a study by Mu et al [5], which reported that adding 20 and 40 mg/kg BW/d grape seed procyanidins to high-concentrate diets improved antioxidant potential of the colonic epithelium and alleviate the local inflammation and disruption of the colonic epithelium in lambs, underscores their potent antioxidant, anti-inflammatory properties, and safety profile. This review systematically synthesizes the impacts of high-concentrate diets on lambs intestinal mucosal barrier function. It then delves into the effects of plant extracts on intestinal physical, chemical, immune and microbial barriers function in lambs. Finally, it explores the underlying regulatory mechanisms of plant extracts in enhancing lambs intestinal barrier function.

## EFFECTS OF HIGH-CONCENTRATE DIETS ON THE INTESTINAL BARRIER FUNCTION OF FATTENING LAMBS

Feeding high-concentrate diets is a common strategy in practical production to increase energy supply levels and improve production performance in lambs. Long-term intake of high-concentrate diets by lambs leads to rapid fermentation of non-structural carbohydrates such as starch in the rumen, generating large amounts of short-chain fatty acids (SCFAs) and producing abnormal metabolites such as histamine, ethanolamine, and lactic acid. This results in a decrease in ruminal pH and metabolic disorders, triggering subacute ruminal acidosis (SARA) [11]. Additionally, when ruminants consume high-concentrate diets, excessive rumen-undegraded starch enters the small and large intestines, causing increased concentrations of volatile fatty acids and lactic acid in the gut, reduced pH levels, and further damage to body health. Excess starch also affects the metabolism of intestinal microbial flora, causing massive death of Gram-negative bacteria and thus releasing large amounts of LPS [12]. LPS can penetrate the gastrointestinal epithelial barrier, be absorbed into the bloodstream along with abnormal metabolites like histamine, and lead to elevated peripheral blood LPS concentrations, thereby inducing systemic inflammatory responses [13]. Therefore, whether the digestive tract barrier is damaged and the extent of damage are critical factors determining whether toxic substances such as LPS can translocate into the bloodstream. The exact site where harmful substances like LPS produced by microbial lysis in lambs fed high-concentrate diets translocate into the blood remains inconclusive. However, the intestinal epithelium composed of a single layer of epithelial cells may be a site where harmful substances more easily translocate, and the intestine is more susceptible to barrier damage and toxin translocation than the rumen.

The intestinal physical barrier, composed of intestinal mucosal epithelial cells, intercellular tight junctions, and the bacterial membrane, is a critical component of the intestinal barrier. It effectively prevents harmful substances such as bacteria and LPS from entering the bloodstream through the intestinal mucosa. A study has shown that feeding high-concentrate diets can cause nuclear rupture of intestinal epithelial cells in goats, widen the gaps in epithelial tight junctions, increase intestinal epithelial permeability, and lead to intestinal mucosal barrier damage [14]. When the intestinal pH drops below a critical threshold, VFA enter non-glandular mucosal cells, disrupting the chloride-dependent Na^+^ transport process and causing necrosis and exfoliation of intestinal epithelial cells [15]. Additionally, excessive LPS released by the apoptotic lysis of Gram-negative bacteria due to increased intestinal acidity also impairs the function of the intestinal physical barrier [16]. The chemical barrier is composed of mucus secreted by intestinal mucosal epithelial cells, digestive juices, and bacteriostatic substances produced by normal resident bacteria in the intestinal lumen. Mucus forms the first barrier between the intestinal lumen and mucosal tissues, keeping bacteria away from intestinal epithelial cells. The abnormal proliferation or deficiency of the mucus layer can lead to related diseases. A study have shown that a decrease in intestinal pH and an increase in free LPS concentration can cause abnormally high expression of intestinal mucosal mucins (MUC), damage the function of the mucus barrier, and trigger diarrhea [17]. Additionally, high concentrations of LPS in the intestine can induce intestinal epithelial cells to produce a large number of reactive oxygen species (ROS) molecules, causing intestinal oxidative damage [18]. Studies have shown that feeding high-concentrate diets can significantly increase the content of malondialdehyde (MDA) in the cecal epithelium of goats, while significantly decreasing the activities of glutathione peroxidase (GSH-Px) and superoxide dismutase (SOD) [19]. In the intestinal mucosal immune system, a large number of immune cells and immune molecules are diffusely distributed within the intestinal mucosal epithelium and lamina propria to exert immune effects. Feeding high-concentrate diets increases the concentration of the quorum sensing signaling molecule C12-HSL in goat colon contents, inhibits the synthesis of MUCs by goblet cells, and disrupts goblet cell homeostasis in the intestine [20]. Additionally, high-concentrate diets can activate the Toll-like receptor 4-nuclear factor kappa B (TLR4-NF-κB) signaling pathway, thereby upregulating the expression of proinflammatory cytokines interleukin (IL)-1β and IL-6 in lamb colonic mucosa [5]. The intestinal microbial barrier is a dynamically stable microecosystem formed by intestinal symbiotic bacteria and the host. Numerous studies have shown that consumption of high-grain diets alters the gut microbiota structure of ruminants, increasing the relative abundances of *Escherichia*, *Clostridium*, *Blautia*, *Turicibacter*, and *Treponema* genera associated with intestinal inflammatory responses [19], while decreasing the relative abundance of *Ruminococcus* that protects intestinal mucosa, thereby disrupting gut homeostasis and inducing metabolic disorders [12]. When Hu sheep are fed a diet with a concentrate-to-roughage ratio of 6:4, colonic microbial fermentation is promoted, and the relative abundances of butyrate-producing bacteria *Anareostipe* significantly increase [15]. Other studies have found that high-concentrate diets reduce gut bacterial richness and diversity in high-yielding dairy cows, with increased relative abundances of *Firmicutes* and decreased *Bacteroidetes* in cecal and colonic contents [21].

**Figure 1:**
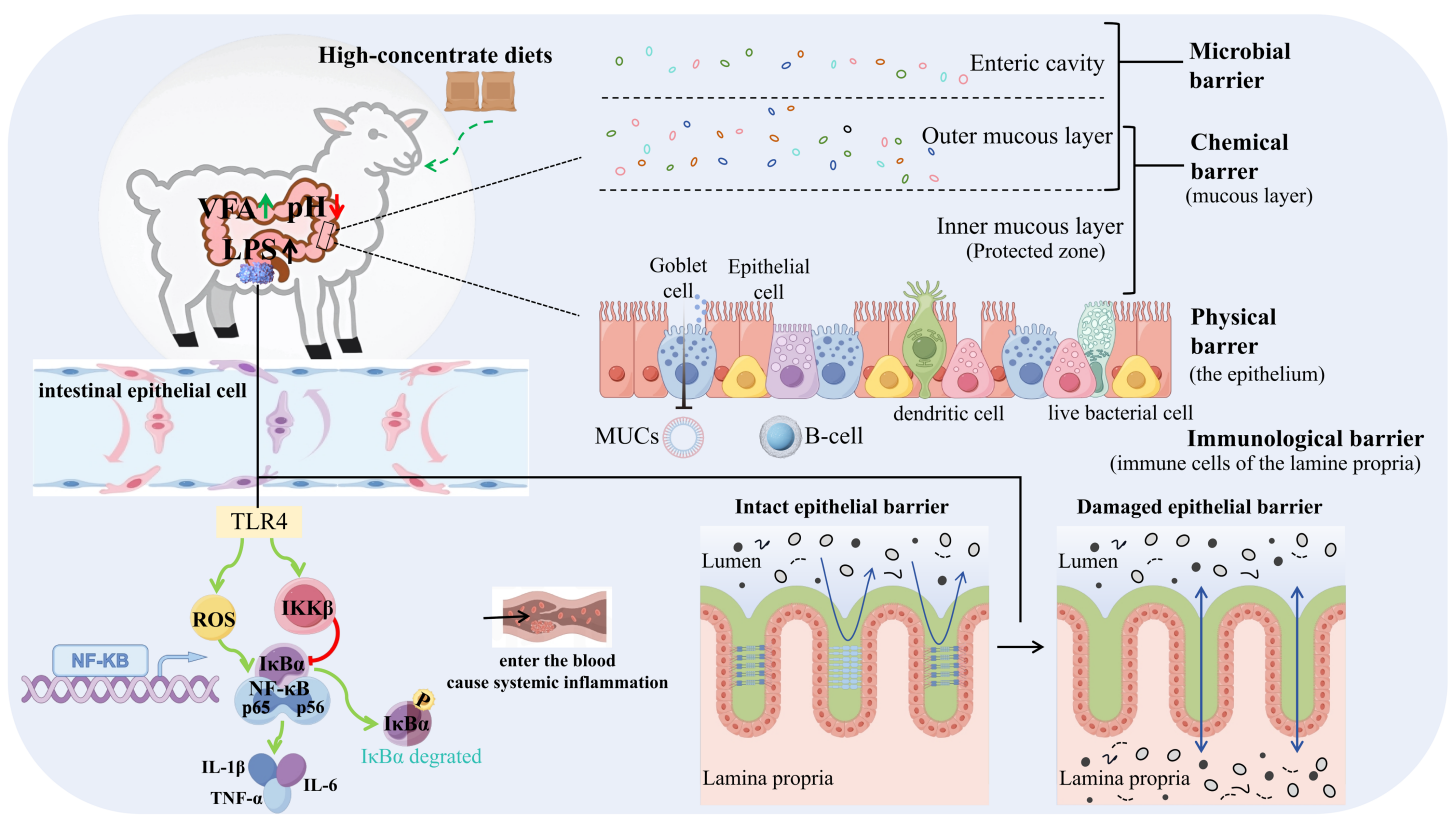
High-concentrate diets damage the intestinal barrier function of fattening lambs. Data from Mu et al [5], Chen et al [7], Ye et al [16]. MUC, mucosal mucins; TLR4, Toll-like receptor 4; ROS, reactive oxygen species; NF-κB, nuclear factor kappa B; IL, interleukin; TNF-α, tumor necrosis factor-α.

In summary, high-concentrate diet intake damages the intestinal epithelial structure of lambs, impairs the functions of intestinal mucosal chemical and immune barriers, alters the gut microecological environment, and ultimately causes intestinal injury (Figure 1).

## THE DEFINITION AND PHYSICOCHEMICAL PROPERTIES OF PLANT EXTRACTS

Plant extracts generally refer to plant secondary metabolites and phytochemicals, including flavonoids, polyphenols, essential oils, and alkaloids. Flavonoid active plant components mainly refer to a class of compounds derived from the parent molecule 2-phenylchromone with a C6-C3-C6 carbon skeleton. According to differences in carbon skeleton types, numbers, linkage positions, and bonding patterns, they can be classified into flavonols, isoflavones, biflavonoids, and other flavonoids. As secondary metabolites of plants, flavonoid active plant components are rich in resources and wide in source, and possess multiple biological functions such as strong anti-inflammatory, antibacterial, antioxidant activities and regulation of animal gastrointestinal health. They also have relatively low toxicity and can be used safely [22]. Essential oil-based plant active components are oily substances containing aromatic molecules extracted and isolated from plants, which are widely used in food, pharmaceutical, and cosmetic industries. They are diverse in types and complex in composition, including terpenoids, aromatic compounds, aliphatic compounds, nitrogen/sulfur-containing compounds, etc., and are characterized by volatility, instability, water insolubility, and special odors. Polysaccharide-based plant active components refer to compounds in plant extracts containing more than 10 glycosyl groups linked by α/β-glycosidic bonds, which possess multiple biological functions such as antioxidant, antibacterial, and immunomodulatory activities. Regarding the antioxidant function of plant polysaccharides, on one hand, the reducing aldehyde hydroxyl and alcoholic hydroxyl groups in polysaccharide molecules can undergo redox reactions with superoxide ion radicals or chelate with metal ions, reducing the excessive production of ROS in cells. On the other hand, plant polysaccharides can enhance the activity of antioxidant enzymes in animal bodies. Plant polysaccharides can also strengthen intestinal barrier function and immune function, and regulate the structure of gut microbiota [23]. Polyphenolic plant active components are a large class of plant secondary metabolites with polyphenolic structural units, widely present in the peels, roots, leaves, and fruits of plants. Currently, more than 8,000 plant polyphenolic components have been identified. Plant polyphenols are generally considered to be tannins and their precursor compounds and polymers with a relative molecular mass between 500 and 3,000. According to different chemical structures, tannins can be divided into two major categories: hydrolyzable tannins and condensed tannins. Hydrolyzable tannins are mainly polygallic acid ester polyphenols, which can be further subdivided into gallotannins and ellagitannins; condensed tannins are mainly condensates of C6-C3-C6 hydroxyflavanols and C6-C2-C6 stilbene monomers. Plant polyphenols exhibit biological activities such as antioxidant, anti-inflammatory, antibacterial, immunomodulatory, anti-aging, and cardiovascular protective effects. They serve as important sources of pharmacologically active ingredients and lead compounds for new drug development, contributing positively to human health. Meanwhile, an increasing number of studies in recent years have shown that plant polyphenols play a positive role in improving animal production performance, alleviating stress in livestock and poultry, and maintaining intestinal health. As a class of natural green antibiotic substitutes with enormous development potential, they possess broad prospects for development [24].

## EFFECTS OF PLANT EXTRACTS ON INTESTINAL BARRIER FUNCTION IN FATTENING LAMBS

### Plant extracts improve intestinal physical barrier in fattening lambs

The intestinal physical barrier is a dynamic and semi-permeable barrier composed of a single layer of columnar epithelial cells, which effectively prevents the free passage of toxic substances in the intestinal lumen through intercellular spaces into surrounding tissues. Intestinal villi are a hallmark of small intestinal tissue morphological development, among which villus height (VH), crypt depth (CD), and the villus-crypt ratio (V/C) are important indicators for evaluating the digestive and absorptive functions of the small intestine and its health status. Complete and long villi are conducive to the full absorption of nutrients and resistance to bacteria. CD is negatively correlated with cell production rate. Deeper crypts indicate lower cell production rates and weaker secretory functions. V/C comprehensively reflects the functional status of the small intestine. Table 1 presents the effects of plant extracts on intestinal morphology and structure in lambs. A decrease in V/C suggests reduced digestive and absorptive functions, accompanied by diarrhea. An increase in V/C indicates a larger intestinal mucosal surface area and stronger digestive and absorptive capacity [36]. Tight junction proteins are primarily composed of cytoplasmic molecules such as claudin, occludin, zona occludens (ZO), and junctional adhesion molecules (JAM). These proteins connect adjacent cells and control the permeability of the intestinal mucosa, serving as one of the main contributors to maintaining the integrity of the intestinal barrier function [37]. Among them: Occludin regulates the paracellular and intercellular diffusion of small molecules. Claudin and ZO control the selective permeability of the intestine by linking to the cytoskeleton [38]. Xu et al [28] reported that supplementing 4 g/kg tea polyphenols to the diet of weaned lambs significantly increased the VH and VH/CD in the duodenum, jejunum, and ileum. Yeast polysaccharides can improve growth performance by alleviating ileal barrier damage caused by weaning stress in lambs, which may be related to yeast polysaccharides upregulating the gene and protein expressions of Claudin1, Occludin, and ZO-1 [30]. Tight junction proteins are regulated by two receptor signaling pathways, TLR4 and epidermal growth factor receptor (EGFR). The former mediates innate immunity and inflammatory responses and inhibits the promoter activation of tight junction proteins [39]. The latter accelerates the synthesis of tight junction proteins by activating the phosphatidylinositol-3-kinase/protein kinase B (PI3K/AKT) and AMP-activated protein kinase (AMPK) signaling pathways to maintain the integrity of the intestinal mucosal barrier [40]. In *in vitro* experiments using porcine intestinal epithelial cells as a model, LPS was found to downregulate the expression of EGFR and its ligands (EGF, hf-EGF, TGF-α). However, the addition of *Portulaca oleracea* polysaccharide (POP) reversed this effect by increasing EGFR expression. Furthermore, when the EGFR inhibitor ZD1839 was co-administered with POP, the protective effect of POP on the intestinal epithelial barrier was abolished [41]. These findings suggest that POP maintains the integrity of the intestinal physical barrier by regulating tight junction protein expression through the activation of the EGFR signaling pathway and the inhibition of the TLR4-NF-κB pathway. In conclusion, plant extracts enhance the physical barrier function of the intestinal tract in lambs through a dual mechanism: regulating intestinal mucosal morphology (e.g., improving villus structure) and upregulating the gene and protein expression of intestinal tight junction proteins (e.g., Claudin, Occludin). These synergistic effects collectively reduce intestinal mucosal permeability and strengthen the physical barrier of the gut.

### Plant extracts improve intestinal chemical barrier in fattening lambs

The intestinal chemical barrier is primarily composed of chemical substances such as bile, digestive enzymes secreted by the gastrointestinal tract, MUC secreted by intestinal goblet cells, and antioxidant enzymes in the intestine. These components exert chemical defense functions in maintaining the balance of the intestinal microecology and intestinal health [42].

Bile is a crucial component of the intestinal mucosal chemical barrier. Bile acids within it can not only alleviate intestinal inflammation but also effectively reduce intestinal pH. In the animal digestive system, an appropriate intestinal pH is of great significance for eliminating pathogenic bacteria. A higher pH promotes the proliferation of pathogenic bacteria and affects the activity of digestive enzymes simultaneously. Mei et al [43] reported that quercetin can influence intestinal mucosal homeostasis by regulating bile synthesis and secretion, the protein expression level of intestinal alkaline phosphatase, and the production of bacteriostatic substances. Zhang et al [44] established a causative link between carvacrol and thymol administration and colitis mitigation using mice as an animal model, primarily through the enhancement of *Bifidobacterium pseudolongum* abundance in the colon, this increase promotes the production of secondary bile acids, particularly hyodeoxycholic acid (HDCA) and 12-ketodeoxycholic acid (12-KCAC), thereby exerting a protective effect on intestinal barrier function.

MUC secreted by intestinal goblet cells, as a major component of the chemical barrier, acts as a lubricant to reduce mechanical damage of intestinal contents to intestinal epithelial cells and prevent the intestinal epithelium from acidic chyme and intestinal pathogens. In Caco-2/HT29-MTX cell co-culture experiments, quercetin and epicatechin were found to promote the gene expression of MUC-17, protecting the intestinal cells from pathogens and bacteria [45]. Serum D-lactic acid (D-LA) and diamine oxidase (DAO) levels are key markers of gastrointestinal integrity. DAO is a highly active intracellular enzyme in the upper villi of the small intestinal mucosa. It can promote cell repair by regulating intracellular ion balance and play a protective role on the intestinal mucosa. D-LA is an indicator of bacterial metabolites and bacterial lysis [46]. Yang et al [31] found in their study that adding 0.3% and 0.5% fucoidan to the diet of weaned goat kids could improve intestinal barrier function, as manifested by decreased serum D-lactate levels and DAO activity. Plant extracts can also interact with digestive enzymes in the intestinal tract to enhance their activity, improving the intestinal absorption function of nutrients. Adding 52 mg/head/d of Oregano essential oils to the basal diet of lambs can significantly increase the activity of amylase in the jejunal mucosa and MUC gene expression [47]. Ma et al [2] studied the potential inffuence of hydrolysable tannin supplementation on intestinal digestive enzyme activity, antioxidant ability and barrier function in fattening lambs. The results showed that 3 g/d hydrolysable tannin significantly increased the trypsin activity in the jejunum and ileum of lambs. In addition, studies have also found that quercetin can promote the absorption of oligopeptides by intestinal villus cells [48].

When animals are under stress, excessive free radicals are produced, causing intestinal damage. Antioxidant enzymes are proteins present in cells that scavenge free radicals in the body. Total antioxidant capacity (T-AOC) is a comprehensive indicator reflecting the body’s antioxidant capacity. SOD can catalyze the conversion of superoxide to oxide in the body. Catalase (CAT) can effectively scavenge oxygen free radicals such as ROS and nitric oxide (NO) in the body. GSH-Px can catalyze the decomposition of hydrogen peroxide in animals. The content of MDA directly determines the degree of lipid per-oxidation in the body, serving as an important indicator for evaluating lipid oxidation damage and indirectly reflecting the degree of intestinal mucosal cell damage [49]. A study showed that dietary supplementation of sainfoin condensed tannins in suckling lambs significantly increased the gene expression of CAT and GPX2 in the ileum. We hypothesize that dietary condensed tannins and their corresponding CT derivatives reaching the intestine may help to reduce local oxidative stress by decreasing ROS production, thereby mitigating immune responses and enhancing endogenous antioxidant defenses [50]. Similar studies found that supplementation of 4 or 6 g/kg dry matter intake (DMI) of tea polyphenols enhanced the antioxidant activity in the intestines of weaned goat kids, Similar studies found that 4 or 6 g/Kg tea polyphenols were found to enhance the antioxidant activity in the intestines of weaned goat kids, significantly reduce the MDA content, and boost the body’s antioxidant capacity [51]. In addition, in other studies, Kudingcha can reduce the levels of NO and iNOS in oxidatively damaged mouse model, and catechins can also reduce the NO content in RAW 264.7 macrophages, inhibiting the production of ROS [52,53]. Nuclear factor E2-related factor 2 (Nrf2), a newly discovered nuclear transcription factor in recent years, can regulate the gene expression of a series of downstream phase II detoxification enzymes such as heme oxygenase-1 (HO-1), NAD(P)H quinone oxidoreductase 1 (NQO-1), glutamate-cysteine ligase catalytic subunit (GCLC), glutamate-cysteine ligase regulatory subunit (GCLM), and antioxidant enzymes such as SOD, CAT, and GSH-Px to enhance the body’s antioxidant defense system. However, recent studies have reported that the Nrf2 pathway not only regulates the body’s antioxidant function by influencing antioxidant enzyme activity but also enhances intestinal barrier integrity by increasing the expression of tight junction proteins [54]. Mu et al [55] found that dietary grape seed proanthocyanidins (20 mg/kg BW and 40 mg/kg BW) increased the gene and protein expression of Nrf2 and HO-1 in colon tissues, reduced local inflammation of colon tissue, and alleviated colon epithelial tissue damage in sheep. This result is in agreement with studies performed in other species, where increase the expression levels of Nrf2 and HO-1 mRNA in the ileal mucosa of broilers supplemented with rutin, it is speculated that rutin may regulate the barrier function of broiler intestines by enhancing the Nrf2-HO-1 signaling pathway [56].

**Figure 2:**
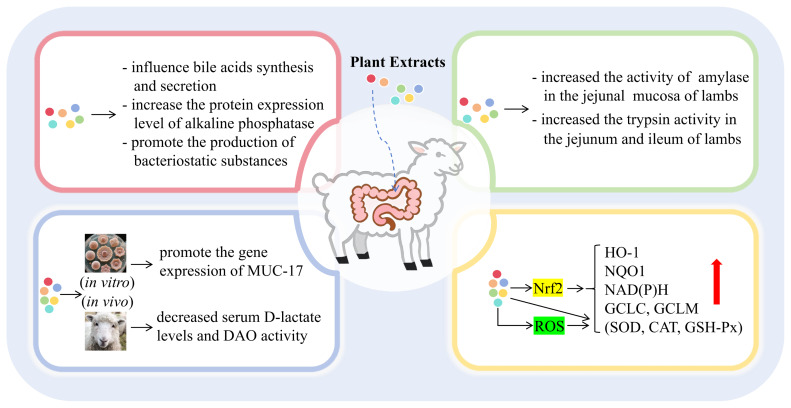
Plant extracts improve intestinal chemical barrier in fattening lambs. Nrf2, nuclear factor E2-related factor 2; ROS, reactive oxygen species; HO-1, heme oxygenase-1; NQO-1, NAD(P)H quinone oxidoreductase 1; GCLC, glutamate-cysteine ligase catalytic subunit; GCLM, glutamate-cysteine ligase regulatory subunit; SOD, superoxide dismutase; CAT, catalase; GSH-Px, glutathione peroxidase. Data from Mei et al [43], Zhang et al [44], Li et al [56], Ma et al [2], Volstatova et al [45], Yang et al [31], Zhao et al [52], Pelegrin-Valls et al [50], Mu et al [55].

The above studies indicate that plant extracts can improve intestinal chemical barrier function through multiple pathways, including regulating the synthesis and secretion of bile, MUC, and intestinal barrier function markers, as well as enhancing the activity of intestinal antioxidant enzymes (Figure 2).

### Plant extracts improve intestinal immune barrier in fattening lambs

The intestinal immune barrier is primarily composed of gut-associated lymphoid tissue (GALT) and antibodies/cytokines secreted by it. These factors not only reflect the immune status of the intestine but also regulate intestinal immune function by modulating local and systemic immune responses. Currently, information on the effects of plant extracts on the intestinal immune barrier function in lambs is limited, with most studies conducted in monogastric animals.

Humoral immunity dominated by secretory immunoglobulin A (SIgA) plays a primary role in the intestinal mucosal immune system. When immune-competent cells in the intestinal mucosal epithelium are stimulated by pathogenic microorganisms, SIgA prevents intestinal microbes and their toxin molecules from attacking the intestinal mucosa. Immunoglobulin A (IgA) can neutralize antigens within cells and return their metabolites to the intestinal lumen, preventing epithelial cell damage caused by pathogen-induced lysis. Immunoglobulin G (IgG), with its complement system, can activate the complement in immune responses to eliminate pathogens [57]. A study reported that dietary supplementation of 0.2 mL/kg DMI tea tree oil (TTO) in Ganxi goats significantly increased IgA secretion in the jejunal and ileal mucosa of lambs. This suggests that TTO can promote local IgA production in the intestinal epithelium after immune stress, thereby preventing bacteria from disrupting the intestinal barrier [58]. In the study using porcine intestinal epithelial cells as a model, both 100 and 500 μg/mL of POPs were found to inhibit the excessive mRNA expression of IL-1β, tumor necrosis factor-α (TNF-α), IL-6 induced by LPS [37].

In the animal body, many physiological and pathological responses, including stress and inflammatory responses, are mediated by specific signaling pathways. The following section will elaborate on the effects of plant extracts on several signaling pathways that govern tissue damage and repair during intestinal inflammation, thereby preliminarily elucidating the protective mechanisms of plant extracts on the intestinal immune barrier function in lambs.

TLRs are recognition receptors on the surface of innate immune cells and other cells, which can recognize stimuli such as LPS, double-stranded DNA of pathogenic microorganisms, single-stranded RNA, and lipoproteins. They play a crucial role in regulating intestinal innate and adaptive immunity [59]. Activation of all TLRs ultimately triggers the NF-κB and mitogen-activated protein kinase (MAPK) signaling pathways, which control the transcription and synthesis of a series of cytokines. As a classical pathway for immune regulation in the body, the NF-κB signaling pathway mediates inflammatory responses by influencing the expression of pro-inflammatory factors such as TNF-α, IL-1β, and IL-6 [60]. Studies have found that jujube polysaccharides can inhibit the activation of the NF-κB pathway by downregulating the expression of NF-κBp65 protein, thereby regulating the synthesis and release of inflammatory factors in mucosal lymphocytes to avoid excessive intestinal inflammatory responses. Additionally, they can enhance intestinal immune function and protect the intestinal barrier [61]. Another study showed that Lycium barbarum polysaccharides can alleviate the damage of inflammatory factors to the intestinal barrier by inhibiting the TLR4/NF-κB signaling pathway and downregulating the mRNA expression of NF-κB, TLR2, TLR4, and MyD88 [62]. In a LPS-induced ileitis rat model, magnolol was found to inhibit the activation of the NF-κB signaling pathway. This action regulated the protein expression and secretion of T cell-derived effector cytokines, increased the mRNA expression level of IL-10, attenuated the gene transcription of TNF-α, monocyte chemoattractant protein-1 (MCP-1), and iNOS, and reduced the contents of TNF-α, IL-6, and IL-1β, thereby alleviating intestinal inflammatory responses [63]. Yang et al [64] supplemented the feed of early-weaned goats with 0.3 g/d Macleaya cordata extract and found that the expression of TLR4 and genes in both the MyD88-dependent pathway (MyD88, TRAF6, and NF-κB) and MyD88-independent pathway (TRAM1, TBK1, and IRF3), as well as downstream pro-inflammatory cytokines (IFN-β, TNF-α, and IL-12B), was downregulated in ileal tissue.

The MAPK signaling pathway plays a critical role in regulating animal intestinal barrier function and the production of pro-inflammatory mediators in the body, making it a recognized effective molecular target for anti-inflammatory therapy. Among its components, extracellular signal-regulated kinase (ERK1) and p38 are key players in the MAPK pathway. Phosphorylation of ERK1/2 and p38 promotes excessive production of inflammatory factors, leading to pathological changes in the intestine [65]. Sun et al [66] found that supplementation with 250 mg/kg quercetin alleviated LPS-induced phosphorylation of ERK, c-Jun N-terminal kinase (JNK), and p38. Ma et al [67] demonstrated that *Pleurotus eryngii* polysaccharides inhibited the activation of the MAPK signaling pathway by suppressing the gene expression of phosphorylated p38MAPK and ERK1/2. These results indicate that plant extracts such as flavonoids and polysaccharides can inhibit the excessive production of inflammatory and chemotactic factors, regulate LPS-induced intestinal inflammation in animals by mediating the MAPK signaling pathway, and improve intestinal health. Additionally, studies have shown that certain plant extracts can regulate the levels of inflammatory cytokines and the gene expression of other mediators involved in the pathogenesis of intestinal inflammation by modulating the Janus kinase/signal transducer and activator of transcription (JAK/STAT) and PI3K/AKT signaling pathways, thereby reducing intestinal inflammation and improving intestinal barrier function [68].

**Figure 3:**
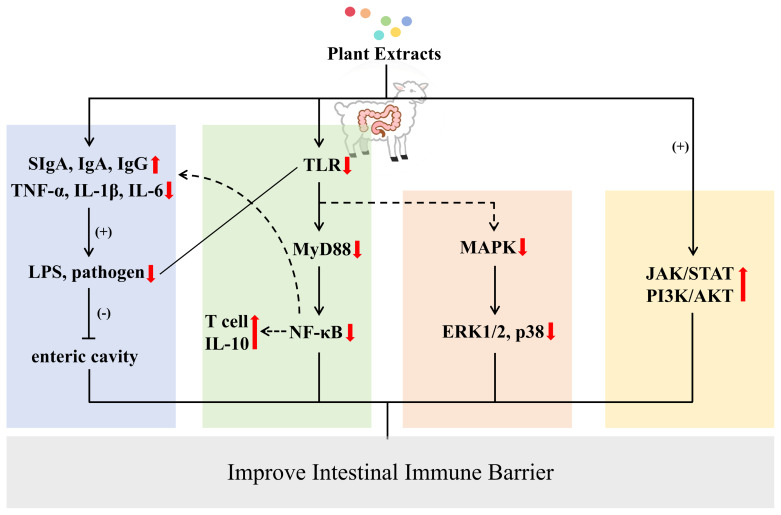
Plant extracts improve intestinal immune barrier in fattening lamb. SIgA, secretory immunoglobulin A; IgA, immunoglobulin A; IgG, immunoglobulin G; TNF-α, tumor necrosis factor-α; IL, interleukin; LPS, lipopolysaccharide; TLR, Toll-like receptor; NF-κB, nuclear factor kappa B; MAPK, mitogen-activated protein kinase; ERK, extracellular signal-regulated kinase; JAK/STAT, Janus kinase/signal transducer and activator of transcription; PI3K/AKT, phosphatidylinositol-3-kinase/protein kinase B.

In summary, during inflammation, inflammatory signaling pathways are activated, leading to increased intestinal inflammation levels and disruption of barrier function. Plant extracts can interact with cell membrane receptors or be absorbed by cells to regulate cell signal transduction, control the expression of inflammatory factors, and protect the intestinal immune barrier (Figure 3). Additionally, the effects of plant extracts on intestinal immune barrier function may help enhance the host’s defense against microbial infections.

### Plant extracts improve intestinal microbial barrier in fattening lambs

**Figure 4:**
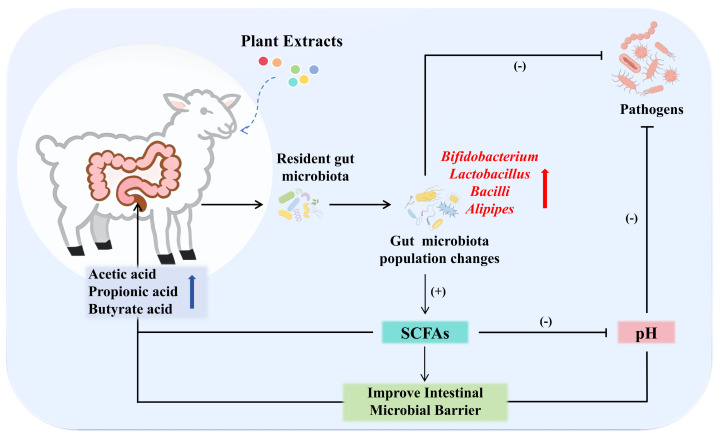
Plant extracts improve intestinal microbial barrier in fattening lambs. SCFA, short-chain fatty acid.

The gastrointestinal tract of lambs is inhabited by tens of thousands of microorganisms, forming an interdependent and mutually restrictive intestinal microbial homeostasis system. Most of these microorganisms colonize the mucosal layer to form a microbial barrier, assisting the mucosal layer in resisting the invasion of harmful microorganisms. Under normal conditions, the intestinal mucosal surface is attached with a large number of anaerobic bacteria, such as probiotics like *Bifidobacterium* and *Lactobacillus*, which can closely bind to the intestinal epithelium through adhesion to form a membrane barrier and prevent pathogenic bacteria from colonizing the intestine [69]. If the intestinal microbial flora is dysregulated, the colonization resistance will decrease, allowing exogenous pathogens to adhere to the intestinal mucosa and induce a series of intestinal diseases, such as intestinal inflammation and diarrhea. In recent years, numerous studies have shown that plant extracts can maintain the balance of the intestinal microbial defense system by selectively promoting the proliferation of beneficial intestinal bacteria, inhibiting the colonization of harmful bacteria, and optimizing the intestinal microecological environment, thereby ultimately preserving the health of the animal intestine and even the entire body. Lee et al [70] found that phenolic compounds extracted from tea leaves exhibit inhibitory effects against pathogenic bacteria such as *Clostridium perfringens*, *Escherichia coli*, *Pseudomonas*, and *Salmonella in vitro*. It is speculated that this may be due to the strong bacteriostatic activity of phenolic substances, which can inhibit the activity of bacterial topoisomerases, thereby suppressing bacterial DNA replication. This antibacterial effect may also be related to blocking nutrient transport in bacterial cells and disrupting the structure of bacterial cell membranes. Additionally, changes in the microbial community structure may increase the content of beneficial SCFAs such as acetic acid and propionic acid, thereby improving intestinal health. Most SCFAs are microbial metabolites. As important energy sources for intestinal epithelial cells and gut microbiota, they not only promote the proliferation and differentiation of intestinal epithelial cells but also improve intestinal mucosal morphology and maintain intestinal mucosal barrier function. SCFAs are considered beneficial metabolites with anti-inflammatory properties and the ability to regulate intestinal immune function. Among them, acetic acid can serve as an energy substrate for peripheral tissues and has good anti-inflammatory and immune-regulatory effects [71]. A study has found that supplementation of 1 g/d mannan-rich fraction can protect the intestinal mucosal barrier by stimulating the metabolic activity of gastrointestinal microbiota in dairy goats, thereby increasing the contents of intestinal acetic acid and total SCFAs [32]. Additionally, it has been reported that supplementation of 8% DM grape pomace (containing tannins) in the diet of Tan lambs can enhance propionate production by gastrointestinal microbiota and related metabolic pathways, promote the production of B vitamins in the rumen, facilitate starch degradation and amino acid biosynthesis in the jejunum, and protect intestinal barrier function [72]. The class *Bacilli* belongs to the phylum Firmicutes. Studies have confirmed that *Bacilli* can induce animals to consume high-fat diets, promote intestinal health, and enhance intestinal absorption function. A study found that supplementation of 2.5 g/kg DMI tea saponin in the diet of Anhui white goats significantly increased the relative abundance of *Bacilli* in the small intestine [26]. As one of the common probiotics, *lactic acid bacteria* occupy a dominant position in the early stage of intestinal microbiota establishment in lambs. An increase in their relative abundance can not only reduce fecal pH and ammonia nitrogen levels but also prevent pathogenic bacteria from colonizing the intestine, thereby improving intestinal health [73]. Inupala et al [74] found that supplementation of garlic extract in the antibiotic-free diet of St. Croix sheep significantly increased the number of *lactic acid bacteria* in feces, indicating that garlic extract has strong prebiotic activity and can protect intestinal health. Studies have shown that the adverse effects of cecal microbiota on the host under chronic stress may be associated with the relative abundance of *Alipipes* [75]. Wang et al [76] found that supplementation of 600 mg/kg DMI Abrus cantoniensis polysaccharides in broiler diets significantly reduced the relative abundance of *Alipipes* in the cecum. Additionally, this study revealed that the relative abundance of cecal *Alipipes* was significantly negatively correlated with ileal SOD activity and ZO-1 expression, and significantly positively correlated with ileal MDA concentration and IL-6 gene expression. These results suggest that Abrus cantoniensis polysaccharides alleviate the negative effects of heat stress on the intestinal tract. Furthermore, it has been reported that resveratrol can also reduce the relative abundance of intestinal *Alipipes* and mitigate the negative impact of LPS on microbiota, showing great potential in preventing intestinal inflammation [69]. This may be because the hydroxyl groups in plant extracts interact with bacterial cell membranes, disrupting membrane structure and causing intracellular components to leak out [77]. These findings collectively reflect the excellent effects and potential value of plant extracts in regulating gut microbiota. They can enhance the relative abundance or quantity of beneficial bacteria, inhibit the proliferation of potential pathogens, and increase gut microbial diversity, thereby strengthening the gut microbial barrier function and ultimately maintaining the health of the animal intestine and the entire body (Figure 4).

## CONCLUSIONS AND PERSPECTIVES

This review first analyzes the effects of high-concentrate diets on intestinal barrier function in lambs, concluding that intake of such diets disrupts intestinal epithelial structure, impairs mucosal chemical and immune barrier functions, alters gut microecological environment, and ultimately leads to intestinal damage. Identifying suitable drugs or feed additives has become a key focus for healthy lamb farming. Plant extracts, characterized by their green, environmentally friendly, and multi-functional properties, have gradually emerged as potential substitutes for feed antibiotics. Numerous studies have confirmed that plant extracts can regulate animal intestinal health by maintaining intestinal barriers, improving gut microbiota, alleviating adverse effects of intestinal inflammation and oxidative stress, and promoting intestinal digestion and absorption. However, current research on the regulation of animal intestinal barrier function by plant extracts mainly focuses on model animals and monogastric animals, with limited studies in ruminants. Additionally, since most plant extracts contain multiple chemical components with strong synergistic effects, the regulatory mechanisms on intestinal functions remain unclear. Therefore, further research is needed to deeply investigate the absorption and metabolism of plant extracts in different animals and physiological conditions, as well as their specific regulatory mechanisms and signaling pathways on intestinal mucosal barriers. Meanwhile, targeted improvements in separation and purification technologies for various plant extracts, more standardized trials to verify their efficacy, and determination of appropriate dosage levels at different growth stages of animals are required. These efforts aim to provide a scientific basis for the development and application of plant extracts as feed additives for livestock and poultry, as well as for healthy lamb farming.

## Figures and Tables

**Table 1:** Effects of plant extracts on intestinal morphology and structure in lambs

Source	Application form	Experiment object	Main function	References
Tannic acid	Basal diet supplemented with 0.2%	Hu male lambs	Up-regulated the mRNA expression of zona occludens (ZO)-1 and occludin protein of jejunum, decreased the crypt depth of jejunum	[25]
Tea saponin	Basal diet supplemented with 0.625, 2.5 g/kg	Weaned lambs	Increased the villi length of duodenum, jejunum and ileum and the ratio of villi length to crypt depth	[26]
Grape seed procyanidins	Basal diet supplemented with 10, 20, 40 mg/kg BW/d	Dorper×thin-tailed Han ram lambs	Improved the protein expression of claudin-1 of colonic epithelium	[5]
Tannins	Basal diet supplemented with 0.1%	Hu sheep	Increased crypt depth of jejunum	[27]
Tea polyphenols	Basal diet supplemented with 2, 4, 6 g/kg	Weaned lambs	Increased villus height, villus height/crypt depth, increased claudin-1 levels in the duodenum, jejunum, and ileum	[28]
Hydrolysable tannin	Basal diet supplemented with 3, 6 g/d	Male Hu sheep lambs	Increased the gene expressions of claudin-1, claudin-4 and ZO-1 in the jejunum	[2]
*Mimosa tenuiflora* (Willd.) hay	Basal diet supplemented 333, 670, and 1,000 g/kg	Santa Ines male lambs	Decreased villi height and surface area of duodenum, increased jejunum villi height, decreased ileum villi height	[29]
Yeast polysaccharides	Basal diet supplemented with 0.5, 1, 2 g/d	Lambs	Enhanced intestinal villus development, increased the villus height-to-crypt depth ratio, upregulated the mRNA and protein expression of Claudin1, Occludin, and ZO-1	[30]
Fucoidan	Basal diet supplemented with 0.1%, 0.3%, and 0.5%	Weaned goat kids	Upregulated the gene expression of ZO-1 and claudin-1 in the duodenum, jejunum, and ileum	[31]
Mannan-rich fraction	Basal diet supplemented with 1 g/d	Xinong Saanen dairy goat kids	Increased ileal villus height and RNA expression of Claudin-1 and Occuldin in the duodenum, ZO-1, junctional adhesion molecules (JAM)-2, and Occuldin in the jejunum, and Claudin-1, JAM-2, and Occuldin in the ileum	[32]
Inulin	18.9% inulin	Weaned goat kids	Improves intestinal mucosal barrier function	[33]
Herbal mixture	100 g/d	Lambs	Increased claudin-1 and occludin levels in the jejunal mucosa	[34]
*Saccharomyces boulardii* yeast wall polysaccharides	Basal diet supplemented with 0.1%, 0.3%, and 0.5%	Crossbred early-weaned lambs	enhanced the intestinal morphology (villus height, crypt depth, and V/C value) of jejunum, ileum	[35]

## Data Availability

Upon reasonable request, the datasets of this study can be available from the corresponding author.
